# Botulinum Toxin and Progressive Pneumoperitoneum in Loss of Domain Ventral Hernias: A Systematic Review

**DOI:** 10.3389/jaws.2024.12650

**Published:** 2024-03-20

**Authors:** Mario Giuffrida, Federico Biolchini, Patrizio Capelli, Filippo Banchini, Gennaro Perrone

**Affiliations:** ^1^ Department of General Surgery, Ospedale Guglielmo da Saliceto, Piacenza, Italy; ^2^ General Surgery Unit, Department of Surgery, Azienda USL—IRCCS di Reggio Emilia, Reggio Emilia, Italy; ^3^ Department of Emergency Surgery, Parma University Hospital, Parma, Italy

**Keywords:** hernia, loss of domain, botulinum, pneumoperitoneum, chemical component separation

## Abstract

**Introduction:** Preoperative progressive pneumoperitoneum (PPP) and botulinum toxin A (BTX) have been used together in the preoperative preparation of patients with loss of domain hernias. This study aims to evaluate the efficacy and safety of the combined use of PPP and BTX.

**Methods:** A systematic electronic search was performed according to the PRISMA criteria. A literature search of scientific articles was conducted up to December 2023. Articles were chosen based on the reference to BTX and PPP in loss of domain ventral hernias with a defect width greater than 10 cm before surgery. The GRADE methodology and the modified Newcastle-Ottawa scale were used to assess the quality of the studies.

**Results:** The research yielded seven articles, with 217 patients analysed in total. BTX was performed 29.5 ± 1.7 days before surgery and PPP was inflated 14.8 ± 5.8 days before surgery. PPP complications were reported in 25.6% of patients, The average reduction of the volume of hernia (VH)/volume of the abdominal cavity (VAC) ratio was 7.6% (range 0.9%–15%). Only 40 patients (18.4%) required a PCS or TAR to repair the loss of domain hernias. The SSI and SSO rates were 17.5% and 26.2%, respectively. No differences in SSI and SSO rates were found between the different repair techniques. The recurrence rate was 5.9% (13/217). Recurrence was significantly higher in patients who underwent IPOM repair than other techniques (*p* < 0.001).

**Conclusion:** BTX and PPP may be useful tools for the management of loss of domain hernias presenting lower SSI and SSO. The combination of BTX and PPP reduces the use of more invasive repair techniques.

## Introduction

Large abdominal hernias with fascial defect diameters greater than 10 cm pose a surgical problem, especially in the presence of loss of domain (LOD). Surgery for LOD hernias can result in complicated fascial closure and an increased risk of high postoperative abdominal pressure, loss of pulmonary capacity and abdominal compartment syndrome [1, 2].

In the last few years several treatment options have been proposed for the management of LOD hernias. In this context, the emerging preoperative progressive pneumoperitoneum (PPP) and botulinum toxin A (BTX) have been used together in the preoperative preparation of patients with LOD hernias [3].

Goni Moreño described for the first time PPP for the stretching of the abdominal wall musculature in 1947 [4]. However, this technique was not without complications, which limited the widespread use of this technique [5, 6].

Preoperative BTX infiltration of the abdominal wall results in decreased tension and elongation of the abdominal muscles, therefore facilitating hernia repair with minimal patient discomfort [7, 8].

BTX induces reversible flaccid paralysis of the lateral abdominal muscles, and has been described as “chemical component separation” [9]. Most of the described techniques involve the administration of BTX 4–6 weeks preoperatively, to increase the compliance of the lateral muscles, followed by PPP for a shorter period of time starting at 2 weeks preoperatively [3–5, 9].

There are varying recommendations for the use of combined PPP and BTX. They are suggested for hernia width greater than 10 cm, LOD greater than 20%–25%, recurrence after component separation, retracted bulky lateral muscles, and anticipated difficulty with midline closure [10].

The objective of this study was to review the published articles on the use of combined PPP and BTX, to assess the efficacy of these procedures in reducing postoperative complications and/or more invasive surgical techniques like component separation.

The PICOS criteria were set as outlined in Table 1.

**TABLE 1:** PICO framework and the question statement.

Criteria	PICOS questions
Population	Patients who benefited from preoperative botulinum and progressive pneumoperitoneum
Intervention	Abdominal wall hernia surgery
Comparison	Combined effect of botulinum and pneumoperitoneum
Outcomes	SSI, SSO, intra-abdominal hypertension (IAH), recurrence
Study	RCT, cohort studies, case-control studies, case series >10 patients

## Subsections Relevant to the Topic

### Search Strategy and Criteria

**FIGURE 1:**
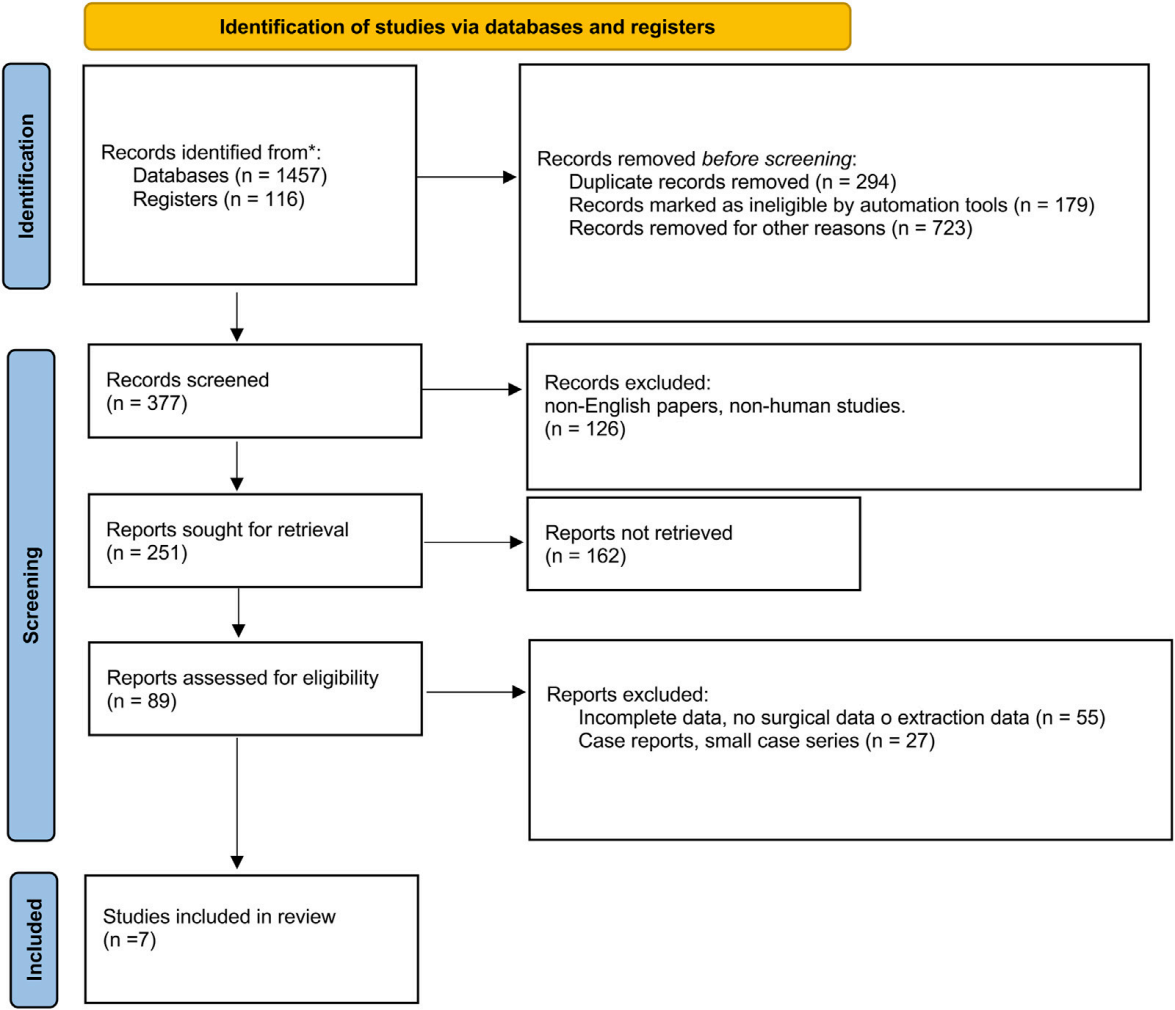
PRISMA 2020 flow diagram.

The International Prospective Register of Systematic Reviews was used to prospectively register the systematic review portion of this study (PROSPERO ID: CRD42024499174) according to the modified PRISMA 2020 guidelines [5] (Figure 1).

All stages of study selection, data abstraction, and quality assessment were carried out independently by two reviewers (M.G. and F.B.). Any disagreements were resolved by consultation with two other reviewers (P.C., G.P.).

### Systematic Review—Information Sources

The systematic review comprised a comprehensive online search of PubMed, EMBASE, MEDLINE and the Cochrane Library for all published articles reporting cases of preoperative botulinum and progressive pneumoperitoneum before ventral hernia surgery until December 2023. The PROSPERO international prospective register of systematic reviews was also queried for published or ongoing reviews of similar scope.

### Systematic Review—Eligibility Criteria

All reported cases of adult patients (age> 18 years) treated with preoperative botulinum and progressive pneumoperitoneum for loss of domain ventral hernias with a defect width greater than 10 cm in the available published literature were included for analysis. Cases were excluded if the studies reported incomplete data or if the studies were not available in the English language or were not performed on humans.

Case reports and small case series (less than 10 patients) were excluded from the final database.

### Systematic Review—Search Strategy

The text word “hernia” was utilised with the headings (MeSH) terms (“botulinum”; “pneumoperitoneum”; “ventral”; “chemical component separation”; “loss domain,” “botox”) using the Boolean operators “AND” and “OR.”

The search was further refined by toggling the “review” and “case report” filter options. The search was expanded using the “related articles” function, and a thorough review of all identified abstracts and citations was performed. Additional eligible cases were identified by screening the bibliographies of all key articles.

### Systematic Review—Data Collection Process

Data were abstracted on the data items of interest (preoperative botulinum and progressive pneumoperitoneum), study characteristics (type of publication, first author, year of publication, aim of the paper), hernia characteristics, botulinum and progressive pneumoperitoneum characteristics and results (dose of botulinum, time and site of botulinum injection, diagnostic exams before surgery, time amount of pneumoperitoneum), treatment characteristics (type of hernia surgery, characteristics) and postoperative outcomes (LOS, complications, Clavien-Dindo classification, SSI, SSO, SSOPI, SSE, recurrence, reintervention, follow-up) [11].

### Systematic Review—Risk of Bias Assessment for This Study 

The level of evidence in the included articles was estimated using the GRADE methodology [12].

The modified Newcastle-Ottawa scale was used to assess the quality of retrospective studies, which consists of three factors: patient selection, comparability of the study groups, and assessment of outcomes [13].

### Systematic Review—Endpoints

This study aims to answer three questions.

- What is the advantage of combined botulinum and progressive pneumoperitoneum for loss of domain ventral hernia?
- Is combined botulinum and progressive pneumoperitoneum safe for patients?
- Does combined botulinum and progressive pneumoperitoneum reduce the need for component separation techniques?

### Statistical Analysis

Data analysis was performed using Jamovi [14].

Descriptive statistical analysis was performed for the main descriptive indices. We did not predict the need for modelling or multivariable analysis. Event rates were reported as percentages (%). Quantitative data were expressed as the mean or median ± standard deviation (SD). Qualitative data were presented as absolute frequencies, relative frequencies, cumulative frequencies, and percentages. Student’s t-test, Mann-Whitney *U*-test or ANOVA were used to compare continuous or ordinal variables. The chi-squared test or Fisher’s exact test, as appropriate, was used for the analysis of categorical data.

### General Characteristics of the Included Studies

The database search identified 1,573 studies. This was reduced to 1,279 after the removal of duplicates. A total of 377 works were screened. After assessment of the abstracts and papers according to the inclusion criteria, seven full-text articles met the inclusion criteria (Figure 1).

A total of 217 patients who underwent PPP and BTX before LOD hernia repair were included in the study. The characteristics of the selected studies are shown in Table 2.

**TABLE 2:** Characteristics of the included studies.

Reference	Study design	No. of patients	Level of evidence	Quality score
Bueno-Lledó 2020 [15]	RCS	100	2B	8
Tang 2021 [16]	RCS	16	3B	6
Elstner 2021 [17]	PCS	39	2B	8
Tang 2021 [18]	PCS	22	2B	8
Yurktap 2021 [19]	RCS	14	3B	7
Tashkandi 2021 [20]	PCS	13	3B	7
Tang 2023 [21]	RCS	13	3A	6

PCS, prospective cohort study; RCS, retrospective cohort study; M, multicenter.

### Population Characteristics

Of the 217 patients analysed, 94 (43.3%) were men and 96 (44.2%) were women. In the remaining 27 (12.4%), sex was not specified.

Obesity was reported in 55/204 (26.9%) patients. Diabetes was reported in 25/104 (24.0%) patients. A total of 42/204 (20.5%) patients were smokers.

Patient characteristics are detailed in Table 3.

**TABLE 3:** Preoperative characteristics.

Reference	No. of patients	Women (%)	Mean age (range or SD)	Mean BMI (kg/m^2^) (range or SD)	Obesity (%)	Diabetes (%)	Smokers (%)
Bueno-Lledó 2020 [15]	100	59 (59.0%)	59.4 (33–81)	—	19 (19.9%)	—	11 (11.0%)
Tang 2021 [16]	16	7 (43.7%)	63.4 ± 9.6 (45–77)	25.7 (22.4–34.7)	1 (6.2%)	3 (18.7%)	2 (12.5%)
Elstner 2021 [17]	39	17 (43.5%)	63.9 ± 19.6 (42–78)	34.2 (21.7–67.9)	24 (61.5%)	12 (30.7%)	18 (30.7%)
Tang 2021 [18]	22	10 (45.4%)	64.9 (44–82)	26.8 (21.3–37.7)	1 (4.5%)	2 (9.0%)	3 (13.5%)
Yurktap 2021 [19]	14	—	65 (28–77)	31.4 (22.7–43.3)	4 (28.5%)	5 (35.5%)	3 (21.4%)
Tashkandi 2021 [20]	13	3 (23.0%)	58 (57–72)	28.7 (26–32)	6 (46.1%)	3 (23.0%)	5 (38.4%)
Tang 2023 [21]	13	—	—	—	—	—	—

BMI, body mass index; SD, standard deviation.

### Indications for PPP and BTX

All articles were assessed for the described indications for the use of BTX and PPP. Hernia width >10 cm was the common indication for each included article.

The authors identified four main themes for the use of BTX and PPP, which are shown in Table 4.

**TABLE 4:** Identified indications for the use of preoperative PPP and BTX.

BTX indications	PPP indications
Hernia width >18 cm [19]	VIH/VAC ratio >20% [15]
VH/VAC ratio >20% [16, 18]	LOD ratio >15% [17]

VH: volume of hernia; VAC: volume of the abdominal cavity; LOD: loss of domain.

### Hernia Characteristics, PPP and BTX Treatment Results

Ventral midline hernia was the most common condition, as it was observed in 145 patients (66.8%), lateral hernias were reported in 46 patients (21.1%), and in 26 cases the location was not reported (11.9%).

The majority of the hernias were recurrent in 56/88 cases (63.6%).

BTX was injected in 6/7 studies (85.7%) at three points on each side of the lateral abdominal wall between the costal margin and the iliac crest lateral to the semilunar line, only Bueno-Lledó et Al [15] used five points on each side as described in a previous study [7]: two points over the mid-axillary line, between the costal margin and the superior iliac crest, and three points over the external oblique muscle.

The average duration of BTX was 29.5 ± 1.7 days before surgery (range 9–119).

The median insufflated air volume for PPP was 9.02 ± 2.7 L (range 3.6–19.5 L).

The average duration of PPP administration was 14.8 ± 5.8 days (range 2–43).

No complications related to the administration of BTX were reported. PPP-related complications were reported in 25.6% (49/191) of patients, and severe complications were reported in five patients (2.6%).

The average reduction in the VH/VAC ratio was 7.6% (range 0.9%–15%).

Heterogeneity was observed at the time of PPP administration, after BTX injection and before surgery among the different papers. Minimal reduction in VH/VAC ratio was observed in studies where PPP was administered within 5.1 ± 2.3 days from BTX injection (*p* = 0.021) and when BTX was administered within 11.6 ± 2.9 days before surgery (*p* = 0.043).

Hernia characteristics before and after BTX and PPP are summarised in Tables 5, 6.

**TABLE 5:** Hernia characteristics before and after BTX and PPP treatment.

Reference	Before PPP + BTX mean (range)			After PPP + BT (range)			VH/VAC ratio reduction
	VH (cc)	VAC (cc)	VH/VAC ratio (%)	VH (cc)	VAC (cc)	VH/VAC ratio (%)	
Bueno-Lledó 2020 [15]	1,517 (615–2,834)	9,322 (4,451–13,001)	29.1 (21.5–36)	2019 (535–3,822)	10,910 (7,780–14,350)	14.1 (3.5–25)	15%
Tang 2021 [16]	1,683 ± 465 (876–2,536)	6,961 ± 2,208 (4,000–12,047)	24.8 ± 5.7 (20.5–50.9)	1817 ± 458 (986–2,768)	10,471 ± 2,122 (6,672–13,909)	17.3 ± 2.7 (13.4–26.9)	8.4%
Elstner 2021 [17]	NR	NR	NR	NR	NR	NR	NR
Tang 2021 [18]	894 ± 640 (216–2,536)	6,692 ± 1931 (3,780–12,748)	13.5 ± 8.5 (3.0–34.0)	1,209 ± 941 (150–3,271)	9,183 ± 2,119 (5,412–15,596)	12.6 ± 8.2 (2.0–27.0)	0.9%
Yurktap 2021^a^ [19]	NR	NR	0.31 (0.12–2.26)^a^	NR	NR	0.33 (0.08–2.00)^a^	6.1%
Tashkandi 2021 [20]	2,800 (1,000–3,300)^b^	6,600 (6,000–9,100)	NR	NR	+4,200 (2,000–5,200)^c^	NR	58.3% (32–82)^c^
Tang 2023 [21]	NR	NR	NR	NR	NR	NR	NR

VH, volume of hernia; VAC, volume of the abdominal cavity; LOD, loss of domain.

^a^ Hernia sac volume/VAC, ratio.

^b^ Median (IQR).

^c^ Median increase in VAC.

**TABLE 6:** BTX and PPP treatment characteristics.

Reference	BTX			PPP		
	Dose	Timing before surgery (days) mean (range)	Complications (%)	Dose (L) (range)	Timing before surgery (days)	Complications (%)
Bueno-Lledó 2020 [15]	NR	38.2 (33–48)	0 (0.0%)	8.8 (4.5–19.5)	12.3 (9–22)	17 (17%)
Tang 2021 [16]	150 UI of BTX	16.3 ± 1.9 (14–21)	0 (0.0%)	6,325 ± 748 (5.4–7.8)	15.9 ± 1.7 (14–19)	2 (13.3%)
Elstner 2021 [17]	500 UI of Dysport^®^	28.7	0 (0.0%)	NR	5.7 (2–16)	23 (60.5%)
Tang 2021 [18]	300 UI BTX	16.0 ± 3.9 (9–23)	0 (0.0%)	6.8 ± 1.5 (3.6–9.2)	18.0 ± 6.8 (9–43)	2 (9.1%)
Yurktap 2021 [19] *	300 UI BTX	45 (28–119)	0 (0.0%)	10.2 (6.4–19.1)	14 (5–43)	5 (35.7%)
Tashkandi 2021 [20]	500 UI of BTAX (Dysport)	33 (27–40)	0 (0.0%)	13 (11–14)	23 (21–30)	NR
Tang 2023 [21]	NR	NR	NR	NR	NR	NR

The mean operative time was 232.5 ± 62.5 min.

Rives technique (RS) was performed 11 times (5.0%), transversus abdominis release (TAR) was performed in 32 (14.7%) patients [22], anterior component separation (ACS) was performed in 70 patients (32.2%), non-specified component separation technique (CST) in 8 (3.6%) patients and laparoscopic intraperitoneal onlay mesh (IPOM) repair was performed in 93 patients (42.8%) and 16 parastomal hernia patients were treated with laparoscopic Sugarbaker (7.3%).

Only 40 patients (18.4%) required a PCS or TAR to repair LOD hernias.

Twenty-six laparoscopic IPOM patients required a minimally invasive external oblique release using a micro-air blade device under visualization [23].

Closure of the midline/defect was reported in 171/174 patients (98.2%) [15, 17, 18, 20].

### Postoperative Outcomes

LOS (length of stay) was 7.78 ± 2.8 days. No differences in LOS were found between IPOM repair and more invasive component separation techniques.

The mean follow-up was 21.2 ± 12.9 months (range 1–100 months).

The surgical site infection (SSI) rate was 17.5% (38/217).

The surgical site occurrence (SSO) rate was 26.2% (57/217). No differences in SSI and SSO rates were found between IPOM repair and more invasive component separation techniques.

The recurrence rate was 5.9% (13/217). Recurrence was significantly higher in patients who underwent IPOM repair (8/93; 8.6%) compared to other techniques (5/124; 4.0%) (*p* < 0.001).

## Discussion

The primary goal of this study was to assess the efficacy of BTX and PPP in reducing postoperative complications and/or more invasive surgical techniques like component separation.

From the synthesis of seven articles, we observed that postoperative complications (17.5% SSI, 26.2% SSO) and (5.9%) recurrences were lower compared with other hernia surgeries for complex LOD hernias without the use of these preoperative techniques [24–26].

Recurrence was reported in only 13/217 (5.9%) patients. We observed a significantly higher recurrence rate in patients who underwent IPOM repair, confirming the results of previous studies where the recurrence rate after IPOM repair was higher in larger defects (defect width >10 cm) [27, 28]. Previous studies reported a recurrence rate of up to 30% [29].

The results of postoperative complications and the recurrence rate of this study may be related to the heterogeneity of the included studies.

We observed limited use of the most invasive repair techniques, with TAR used in only 14.7% of patients and ACS used in 32.2% of patients. Less invasive techniques (RS and IPOM) were used in 49.4% of the patients.

In this context PPP and more recently BTX have been introduced for the management of LOD hernias.

The results of the present study suggest that the use of PPP and BTX is an additional value in the preparation of LOD hernia repair thanks to the reduction of the hernia-to-abdominal-cavity volume ratio and the gain of muscle length in the axial plane resulting in a significant reduction in hernia defect size and increasing defect closure rates [3, 17, 30, 31].

LOD hernia represents a chronic muscle retraction and contracture that cannot be repaired with a simple fascial closure technique and could lead to potential problems such as abdominal compartment syndrome, ventilatory restriction, and an elevated risk of hernia recurrence [9, 32].

PPP was used for the first time in 1947 [4] and since then its application has been controversial due to the limited availability of literature evidence. There is no consensus in the literature about the amount of air, frequency and duration of insufflation that must be administered [5, 6, 33, 34]. Furthermore, PPP is associated with several complications, and we reported a complication rate of 25.6%, with severe complications reported in five patients (2.6%), versus no complications related to BTX injection.

In this study we observed a wide variability in PPP management. The median amount of insufflated air was 9.02 ± 2.7 L with a range of 3.6–19.5 L. The time of PPP administration ranged from 2 to 43 days before surgery. Also, the time from BTX to PPP was different among the different studies, with heterogeneity that may influence the potential benefits of BTX and PPP. We found that PPP performed within 5.1 ± 2.3 days of the BTX injection led to a smaller reduction of the VH/VAC ratio.

Despite the wide variability in PPP management, several advantages have been reported. PPP can perform pneumatic adhesiolysis prior to repair, allowing the spontaneous reduction of the hernia contents and the amount of dissection during surgery with a benefit for diaphragmatic function [35–37].

In the past decade BTX has been introduced into the management of complex ventral hernias.

BTX facilitates the closure of fascial defects without respiratory compromise thanks to the increase in abdominal volume and the reduction of abdominal wall tension. The effect of BTX is maximal at 2 weeks and lasts for 6–9 months [38]. Two protocols for BTX injections have been described: the three-point technique and the five-point technique [7, 39]. In our study the three-point technique was used in 85.7% of the works examined and the five-point technique was used in only one study [15]. BTX was injected with a range of 9–119 days before surgery. We found that BTX administered within 11.6 ± 2.9 days before surgery led to a lower reduction of the VH/VAC ratio.

Despite the general suggestions gathered in the present study, a more detailed analysis of the role of BTX and PPP is needed. We have taken stock of what has been done so far.

Our paper had several limitations that should be taken into account. First, although there was no evidence of publication bias, among the included studies four were retrospective studies and three were prospective cohort studies. This increased the risk of bias due to inadequate randomization and blinding. Second, this study cannot provide comparative results because all articles lacked a comparison group.

Studies with small numbers of patients were included, and often no prospective protocol for data collection was described. The inclusion of consecutive patients was not described in all studies.

The included studies also reported different hernia characteristics with wide heterogeneity in treatments.

A further limitation is that the number of recurrences may be biased, as many studies did not have a standardised follow-up protocol and had different follow-up times.

Nevertheless, the results of this study are encouraging. BTX and PPP may be useful tools in the management of LOD hernias. BTX and PPP showed lower SSI and SSO compared to hernia surgery without these techniques and also reduced the use of more invasive repair techniques.

However, when these techniques should be used remains a matter of debate. The indications for the use of BTX and PPP are not well understood and often depend on CT measurements and much more on surgeon preference.

## Abbreviations

PPP, preoperative progressive pneumoperitoneum; BTX, botulinum; LOD, loss of domain; SSI, Surgical Site Infection; SSO, Surgical Site Occurrence; IAH, intra-abdominal hypertension; RS, Rives-Stoppa technique; ACS, Anterior Component Separation; CST, Component Separation Technique; TAR, Transverse Abdominal Release; IPOM, Intraperitoneal Onlay Mesh; LOS, Length of Stay; VH, Volume of hernia; VAC, Volume of the abdominal cavity.
